# An exploratory study to develop a practical ethical framework for reproductive health research 

**Published:** 2013-01

**Authors:** Tahmineh Farajkhoda, Robab Latifnejad Roudsari, Mahmoud Abbasi

**Affiliations:** 1 *Department of Midwifery, School of Nursing and Midwifery, Shahid Sadoughi University of Medical Sciences, Yazd, Iran. *; 2 *Nursing and Midwifery School, Shahid Beheshti University of Medical Sciences, Tehran, Iran.*; 3 *Department of Midwifery, School of Nursing and Midwifery, Patient Safety and Health Quality Research Center, Mashhad University of Medical Sciences, Mashhad, Iran.*; 4 *Shahid Beheshti Medical Law and Ethics Research Center, Shahid Beheshti University of Medical Sciences, Tehran, Iran.*

**Keywords:** *Reproductive health*, *Research*, *Ethical framework*

## Abstract

**Background: **Research in reproductive health (RH) has been located in the core of women’s health research. Providing accurate information through conducting scientific and controlled research is essential, but increased number of research in the world especially in developing countries in RH area in order to introduce advanced technologies has been resulted in much unethical, illegal and abusive research on women, which needs particular attention to ethical issues by the practitioners who are involved in RH research.

**Objective:** This study was conducted to develop a practical ethical framework for RH research.

**Materials and Methods:** 45 expert academics and clinicians in various disciplines included in a three rounds Delphi study through purposeful sampling method. In round 1 Delphi data were gathered using open-ended questions by e-mail and answers were analyzed by conventional content analysis and the findings merged and validated with the results of a thorough literature review. Face and content validity index were determined in round 2 Delphi and consensuses were attained in round 3.

**Results:** Emerged categories were 1) management of the research process 2) protection of participants’ rights 3) third party consent 4) gender sensitive research and 5) conflict of interest.

**Conclusion:** This study has provided a practical ethical framework according to the socio-cultural context of Iran for all practitioners who are involved in research on women. Adherence to this framework may protect practitioners against unethical and illegal lawsuits and help them to respect their clients’ reproductive rights.

## Introduction

“Research” is one of the most important professional role of practitioners in their professional life; because advancement in medicine depends on research. 

Furthermore, introducing new technologies or modern diagnostic and treatment methods, and also improving public health is remarkably linked to the research endeavors (1-3). However, it should be considered that increased number of medical research without particular attention to the ethical standards may be detrimental for the patients (4, 5). The right of women and men to enjoy the benefits of scientific progress shows high priority of conducting robust reproductive health (RH) research based on ethical considerations, which their results can be used in safe practice (6, 7). 

Despite improvement of global health, the RH profile of many developing-countries populations has remained unsatisfied and poor. Existing gap in quality of RH services and professionalism due to ethical dilemmas is obvious especially in the care of vulnerable groups (8, 9). Rapid changes related to the introduction of new health technologies like assisted reproductive technology (ART), prenatal screening and treatment have encountered practitioners with new legal and ethical challenges in health services delivery to the clients (4, 9-11). 

Also violation of participants’ right by inappropriate use or abuse them in medical research have been reported. Primary responsibility of practitioners is providing guaranteed health for the clients, whereas the researcher’s primary responsibility is the generation of knowledge, which may or may not accompany with the research participants’ health (1, 6, 7). Ethical codes for research are useful tools to control ethical issues in research process (2). 

Although the specific national ethical guidelines for biomedical research have been introduced in Iran and research in bioethics are growing since last decade, but the “Six Ethical Codes for Research” in Iran have not enough clarity, comprehensiveness and precision (4, 12). Mohammad Nejad *et al* (2011) have stressed expertise debate for identifying the barriers in order to put into practice the Bill of Patients’ Rights and professional code of ethics in many realms including research area in Iran (13). 

Also Khodakarami and Jannesari have reported that developing professional code of ethics in RH area is a necessity in Iran (14). In view of lack of medical codes of ethics (including research-related codes) for various groups of medical professions including RH practice, it seems necessary to develop an ethical framework for RH professionals due to the sensitivity of research on reproductive issues. 

Considering that women's rights should be guaranteed in scientific research, this study was conducted to develop an applicable ethical framework for RH research in Iran. We hope that this could introduce an ethical framework for practitioners in research on human reproduction to guarantee their professional rights and the clients’ rights too.

## Materials and methods

A large sequential exploratory mixed method study including a Delphi study in relation to developing codes of ethics in RH was carried out between March 2010 and August 2011 in four medical sciences universities in Iran (15). The ethics committee of Shahid Beheshti Uuniversity of Medical Sciences confirmed conducting the study project. This article reports the findings of the first phase of the study in which a three rounds Delphi was used. 

Delphi is considered as a valid and scientific method in order to provide valid and comprehensive data concerning an important problematic issue or achievement of consensus regarding a matter which it needs scientific experts judgment through combination of qualitative and quantitative processes. Sample size in Delphi study depends on homogeneity of expert panel, disciplines diversity and aims of the study. An average range between 10 to 50 experts has been recommended (16). 

For obtaining scientific and valuable data from stakeholders and practitioners in RH services, forty-five academics and clinicians from four universities including Tehran, Shahid Beheshti, Isfahan and Mashhad universities of medical sciences were chosen through purposeful sampling as expert panel members. They were selected from various disciplines based on their expertise in relation to RH care and also according to the aims of study. They consisted of five Obstetricians and Gynecologists, 15 RH specialists, 10 Medical Ethicists, two General Practitioners (GP), six Midwives, two family health providers, three lawyers and two clergymen with at least three years of work experience. 

In the first round of Delphi (qualitative part of research) a questionnaire consisted of various open-ended questions regarding conducting a proper research, participants’ rights and problematic issues in medical research on women was disseminated to the experts through e-mail. Returned answers were analyzed using conventional content analysis. The findings were merged with the results of a thorough literature review. In the second round (quantitative part) a primary draft of codes of ethics was delivered to the experts via e-mail and they were asked to rate the importance of the statements in order to evaluate the face validity, and also to rate statements’ relevancy, clarity and simplicity to measure content validity index (17). 

At the end of the second round, several number of expert panel members participated in a face-to-face dialogue in order to choose appropriate and accurate writing method of each statement based on the religious, legal and ethical considerations in Iranian culture. In round 3 Delphi (quantitative part), after receiving returned answers to the questionnaires final consensus of expert panel members was considered as ethical framework on research in RH services.

## Results

Twelve male (26.66%) and 33 female (73.33%) experts participated in the study. Their ages ranged from 37 to 58 years old (mean age: 42.52±5.46). Mean length of their work experience was 15.76±5.20 years. Thirty eight participants (84.4%) were faculty members. All experts in round 1 Delphi answered the questionnaire completely (response rate was 100%). Their responses were set a draft of codes of ethics regarding research in RH with adequate data to structure the second round questionnaire. 

The results of study regarding research in RH were arranged into five categories including management of the research process, protection of participants’ rights, third party consent, gender sensitive research, and conflict of interest. These five main categories were divided to several subcategories in order to explain professional code of ethics on research in RH (Table I).

**Management of** **the research process**

Management of the research process was addressed by the majority of the experts. They believed that it is necessary for conducting a systematic and accurate research. One medical ethicist mentioned: “Practitioner should be familiar with the scientific process of conducting a research”. Saving and storage of research information was one of the issues that many of the experts addressed it. 

A GP declared: “Research information should be kept in confidence even after terminating the research”. Monitoring of research process was stressed by several experts. One Obstetrician and Gynecologist believed: “Research process in all stages of research should be observed, monitored and evaluated in order to respect professional integrity based on the research proposal”. Respect to the rights/interests of research institute and researcher was another important point stressed by many of the experts. A lawyer stated: “Research institute and researcher’s interest should be protected in a legal framework through all research stages from designing to publication”.

**Protection the rights of participants**

Protecting the rights of participants was declared as one of the main category by the majority of experts. Experts believed that the rights of participants should be respected in all stages of research. One RH specialist in this regard said: “The national codes of ethics of medical research on human participants should be respected by the researchers”. Paying attention to the essential ethical principals was also emphasized by many of the experts. A medical ethicist stated: “Essential ethical principals such as autonomy, beneficiary, non-maleficent and justice should be applied in research”. Informed consent issue was introduced as a key element by the experts. A family health provider in this regard believed: “Participants should involve in the research and announce their freely informed consent after getting full awareness about research aims without any force or coercion”.

**Third party consent**

Consent by third party was also stressed by many of the experts as a challenging issue. Obtaining consent from incompetent persons regarding participation in the research was mentioned by the majority of experts. A medical ethicist declared: “In the case of research on mental incompetent persons, informed consent should be obtained from their legal representatives”. Several experts pointed to getting informed consent for participation of minors in research. 

An Obstetrician and Gynecologist emphasized: “In the case of research on minors, informed consent should be obtained from their parents or their legal representatives; in addition minors’ agreement should be obtained if it is possible”. Many of the experts believed that husband’s authorization and agreement should be given for his wife’s participation in the research in some circumstances that shared decision making is needed. One RH specialist stated: “Practitioners should obtain husband’s authorization for his wife’s participation in the research before involving her in the research”. 

**Gender sensitive research**

Issues regarding gender sensitive research were one of the most important matters mentioned by the majority of experts. They believed that women’s reproductive system should not tolerate increased burden because of research. One Obstetrician and Gynecologist stated: “Women should not be involved in the research only for their reproductive system status”. Also many experts indicated that particular health status of vulnerable women should not be considered as a factor for participating in research. 

A RH specialist stressed: “Infertility, malignancy, poor socio-economic status and also other impaired health status of women should not be a reason for including them in the research without their agreement”. Considering women’s situation in various stages of their life was mentioned by several expert panel members. A midwife emphasized: “Practitioner should check woman’s pregnancy status before involving her in some studies”. Taking into account pregnant woman and her fetus as a unique unit in research was declared by many experts. One medical ethicist stated: “Practitioner should keep the pregnant woman and her fetus safe as a unique unit through conduction of research”. 

**Table I:** All categories and subcategories of the study

**Management of the research process** **Practitioner should:** Be familiar with the scientific and systematic process of conducting a research. Conduct the research after gaining ethics committee approval. Keep the mutual interests of the research institute, sponsor and researcher in a legal framework through all research stages from designing to publishing. Observe, monitor and evaluate the research process in all stages of the research based on research proposal in order to respect professional integrity for avoiding occurrence of any wanted or unwanted bias due to personal or institutional interests. Keep research information in confidence even after its termination. Select an appropriate participant according to research objects. Publish the results of the research in order to provide better health for human beings. Complete peer review process of others in a fairly manner when it is needed. Avoid any duplication of findings.
**Protection the rights of participants** **Practitioner should:** Respect approved national codes of ethics in medical research regarding human participants derived from international ethical guidelines. Inform the participants regarding study aims before involving them in the research. Involve the participants in the research for fulfilling the best to benefit for them, avoid any harm to them and compensate their redress even in educational fields. Obtain free informed consent without proxy from the participants without any coercion or abusive behavior based on professional trustworthiness after giving necessary and adequate information regarding benefits, side effects, and risks of participation in the research. Respect the right of privacy and confidentiality of the participants. Respect the right of the participants to exclude from the research in any time that they want. Respect the right of the participants to know the results of the research. Never deprive necessary and appropriate care and treatment of whom rejected participation in the research. Pay attention that participation in the research should not be a barrier for giving appropriate care and treatment.
**Third party consent ** **Practitioner should:** Pay more attention to minors or mentally incompetent participants. They should involve in the research if the research provide the best benefit for them. Free informed consent should be obtained from their legal representatives. Obtain husbands agreement in addition to women’s informed consent in specific research such as research on pregnant women.
**Gender sensitive research** **Practitioner should:** Pay more attention to the age and specific health needs of the participants in the research. Infertility, malignancy, poor socio-economic status and also other impaired health status of women should not be a reason for including them in the research without their agreement. Conduct the research without any discrimination based on sex, age, ethnicity, religion, socio-cultural and health status. Never involve the women in the research only based on their reproductive system in order to avoid increasing burden of research on them. Pregnant women should involve in the research as a unique unit if the research provide the best benefit for their fetuses and them.
**Conflict of interest** **Practitioner should:** Diagnose and divulge the conflict of interest when it arises. Manage and control the conflict of interests of the participants, family and public interests versus personal or institution interests accurately. Consult with experts of professional societies if resolving the conflict of interests is impossible. Avoid any inappropriate or abusive relationships with the client in order to involve them in the research.

## Discussion

The main categories emerged from this research were respect to the research process, protection of participants’ rights, consent by proxy, gender sensitive research, and conflict of interest.

Management of the research process mentioned as a key element in medical research by most of the experts. They believed that controlling of integrity in medical research is very important. Respecting the professional integrity including accuracy, honesty, and truthfulness, ethical principles, national laws, institutional regulations, and scientific standards in the all stages of research including planning, designing, conducting, collecting, analyzing and interpreting of data, reporting and publicizing the research results has been emphasized by different associations (18, 19). 

The findings of this study highlighted consideration of ethical standards in medical research. The experts believed that researchers are accountable for their great responsibility. Lapses in ethical standard or technical incompetency can produce unacceptable findings and can threat the professionalism (7, 18). Both of principal researcher and persons involved in the research activities should perform their duties and commitments to sponsors and organization and should keep the confidential nature of the research and its results (19). The researchers should respect accurate data gathering without any bias, fabrication, falsification and respect acquisition, management, sharing, ownership, authorship, copyright laws, editorship, peer review process and plagiarism too (20, 21). 

According to the findings of this study, stressing on participants’ rights protection and their safety in research has been addressed by the majority of experts. Also based on Islamic-Iranian culture human dignity and participants’ rights protection was introduced as a cardinal principal in medical research. Worldwide increasing awareness among right to health and emphasizing on Nuremberg Code, Declaration of Helsinki and all professional codes of medical ethics have located the monitoring of participants’ safety, rights and welfare in the core of Data and Safety Monitoring Committee (DSMC) activities (22, 23). 

In this study, the experts believed that ethics committees have an important role to control participants’ safety through conducting research. Ethics Committees’ competency to respect high enough standard in the research is a problematic issue, thus developing a network of Research Ethics Committees to provide needed knowledge to better protection of the participants’ rights has been suggested (24). Raising the rate of clinical trials in low and middle income countries, the application of principles of ethical research, including respect for participants' integrity and autonomy, obtaining informed consent, providing appropriate participants' information, post research commitments to participants and developing of clinical guidelines have been obligatory (25-28). 

In this study experts stressed that getting informed consent from participants is essential. Obtaining appropriate informed consent using comprehensible language to research participants including to make clear the purposes of the research and unexamined procedures, irritations and risks, benefits, necessary instructions and answering participants' questions, limitations of confidentiality, being free to withdraw the consent at any time has been stressed (29). 

According to Bindra and Kochhar (2010) only 18% truly informed consent was obtained from the participants in clinical trials in India. In addition, respect to participants’ autonomy for involving in the research was 86% and giving enough information to participants was determined 68%. They also reported that 40% of the investigators believed that illiteracy was a negative factor in informed consent process, but low social class and female sex has no impact (5). 

The expert panel in this study believed that deviation from standards should be reported by the researchers. When any deviation from acceptable standard practices or any unwanted adverse effects is emerged through conducting the research, researchers should disclose them and consult with professional expertise to protect the rights of research participants (19). 

Third party consent was highlighted by several experts in the current study. According to the study findings informed consent should be given from incompetent persons’ representatives. Participation of incompetent persons including adolescents under the age of legal majority and mental disabled persons should be restricted to the cases that the study accompanies with considerable advantages for them. Also it should be limited to the situations where the study conduction is impossible on other population and existing knowledge cannot solve their RH problems. In these circumstances after giving appropriate information and obtaining informed consent from their parents or their legal representatives, their other rights should be respected (7, 30). 

In this study, the experts believed that husbands’ authorization for participation of their wives in medical research is an important matter according to the particular cultural context of Iranian society. Husbands’ authorization for participation of their wives in research mentioned as an important issue by experts. Although husbands’ authorization for participation of their wives in research violates participants’ rights, in rare circumstances including particular socio-cultural status, legal requirements, research on pregnant women and their fetuses and also nursing women, husbands’ agreement is necessary (6, 30.(

Gender sensitive research which was pointed out by the majority of expert panel members is one of the most problematic issues in RH research. They believed that research should not be conducted on women only based on their reproductive issues and the benefits of research should be obvious. Gender sensitive research refers to how does the technology, intervention or behavior fit in woman’s and men’s lives. Department of Reproductive Health and Research (RHR) of United Nations (UN) confirmed that intervention or research should not accompany with gender inequality (30). 

In this study the experts emphasized on avoiding all discrimination through conducting the research. Therefore ethical principles emphasis that research should be conducted without any discrimination based on sex, age, ethnicity, race, religion, socio-cultural and health status of the participants. Women are particularly vulnerable to personal harm or discrimination because of existing unequal power relationships in the society that may act as a barrier against women's self-determination (6, 7). 

Research on women in reproductive age was introduced as a problematic matter in this study by expert panel members. In ethics literature, research on women of reproductive age has been addressed with many significant concerns too (6, 11). Increasing research on prenatal screening and treatment methods accompany with many serious ethical challenges. Also a variety of modern ARTs are frequently introduced in clinical practice without an appropriate evaluation of their effectiveness or safety. According to The European Society of Human Reproduction, research on these topics should be conducted through well-designed research and long-term follow-up studies (7, 31). 

Health status of women such as women with malignant diseases was emphasized by expert panel members. Involving participants in cancer clinical trials usually has been associated with arguments for all investigators. Women with cancer have a greater risk for participation in inappropriate research because they may seek every tool for treating their disease (6, 32). In recent years complementary medicine are frequently used for cancer care in the Middle East without enough approved outcomes (33). Thus, in the new trends, practitioners’ instruction should be carried out through appropriate instruction regarding participants’ safety and ethical concerns (2, 32). 

Conflict of interest was declared as a major problematic issue by the great numbers of experts. They believed that it is an inevitable matter in research but researchers should have enough skills to manage the issue of conflict of interest appropriately. Since research has a collaborative and interdisciplinary nature therefore it involves several individuals from various disciplines and various organizations, all of them should collaborate with respect to the interest and trust (18). According to the study findings, the expert panel members believed that disclosure and consulting with related professional societies is an ethical and logical approach against conflict of interest. 

Cook and Dicknes (2000) emphasized that the best protection against conflict of interest is full and timely disclosure and consulting with professional experts too (6). Experts in this study stressed on avoiding any non-professional relationships with the participants and their relatives in research. Practitioners should avoid any inappropriate professional relationships or abusive relationships including emotional, sexual or financial relationships with the clients, family or their relatives in order to involve them in the research. When the conflict of interest arises they should put participants’ interest above of personal or organizational interest based on legal and ethical framework (18). 

## Conclusion

Everyday ethical concerns raised in women’s health care shows necessity of acting responsibly and ethically and practitioners should be who know, understand, and practice in an ethical manner at all times. Medical practitioners must provide accurate information to insure a high standard of health for the populations through conducting scientific and controlled research. Therefore the most commonly factor of conflict of interest is a financial issue, giving invalid and unqualified information for the reason of increased financial gain which is unethical and threats the health of populations and professionalism too. 

This study has suggested and introduced practical recommendations for all who involve in research on women. It may protect them against unethical and illegal lawsuits and respecting women’s reproductive health rights and their welfare too. As the primary commitment of RH practitioners is to serve women’s reproductive health and wellbeing, so engaging in research is an important activity to address the core problems of women’s health, providing essential knowledge and applying research findings to the policies and programs related to RH.
